# Genetic variation for the duration of pre-anthesis development in durum wheat and its interaction with vernalization treatment and photoperiod

**DOI:** 10.1093/jxb/eru170

**Published:** 2014-04-30

**Authors:** Gavino Sanna, Francesco Giunta, Rosella Motzo, Anna Maria Mastrangelo, Pasquale De Vita

**Affiliations:** ^1^Unit of Agronomy, Field Crops and Genetics, Department of Agriculture, University of Sassari, Via De Nicola 1, 07100 Sassari, Italy; ^2^CRA Cereal Research Centre (CRA-CER), S.S. 16, km 675, 71122 Foggia, Italy

**Keywords:** Durum wheat, leaf number, phenology, phyllochron, pre-anthesis, QTL.

## Abstract

A recombinant inbred durum wheat population was grown under three contrasting regimes: long days following vernalization (LDV), long days without vernalization (LD), and short days following vernalization (SDV). The length of several pre-anthesis stages and the number of leaves and the phyllochron were measured. Different groups of genes were involved in determining the phenology in the three treatments, as demonstrated by a quantitative trait locus (QTL) analysis. The length of the period required to reach the terminal spikelet stage was correlated with the time to anthesis only in the case of LDV- and LD-grown plants where the timing of anthesis depended on the final leaf number. However, for SDV-grown plants, anthesis date was more dependent on the length of the period between the terminal spikelet stage and anthesis and was independent of leaf number. The involvement of the phyllochron in determining the duration of pre-anthesis development was also treatment-dependent. QTL mapping of the various flowering time associated traits uncovered some novel loci (such as those associated with the phyllochron), in addition to confirming the presence of several well-established loci.

## Introduction

Flowering time is an important determinant of grain yield (Reynolds *et al.*, 2009), and its manipulation is a common breeding target. Studies of the genetic determination of flowering time in wheat have demonstrated that it is controlled by at least 20 genes, scattered over the whole genome (Snape *et al.*, 1996; Koornneef *et al.*, 1998); these genes have been classified according to whether they respond to vernalization or to photoperiod or whether they confer earliness *per se* (EPS; Worland, 1996; Law and Worland, 1997; Laurie *et al.*, 2004). Much less information, however, has been gathered concerning the genetic basis of the duration of the various pre-anthesis stages of spike growth (Borràs-Gelonch *et al.*, 2011) and the extent to which these vary in a genetically determined manner remains unclear (Gonzáles *et al.*, 2005a; Whitechurch *et al.*, 2007; Borràs-Gelonch *et al.*, 2011). The period during which most of the growth of the wheat spike occurs coincides with the stem elongation stage, so lengthening the latter can be expected to increase the size of the spike and, by implication, also the number of potential grains that are set (Halloran and Pennell, 1982; Slafer *et al.*, 1996; Slafer and Whitechurch, 2001). According to Fischer (1983), the period between the emergence of the penultimate leaf and anthesis is the most critical stage of the spike’s growth. Thus, the ability to fine-tune crop phenology offers some potential to increase spike fertility (Fischer, 2011; Foulkes *et al.*, 2011; García *et al.*, 2011).

The main stem of the wheat plant develops via the accumulation of primordia at the stem apex and their subsequent differentiation into either vegetative (leaf) or reproductive (spikelet) structures, thereby providing a mechanism whereby the duration of the various growth stages can vary (Jamieson *et al.*, 1998). The inverse of the rate of leaf appearance (the ‘phyllochron’) is about double the inverse of the rate of primordium production (the ‘plastochron’) (Kirby, 1990). The period over which primordia are initiated depends both on the plastochron and the number of primordia actually initiated, while the period over which leaves emerge depends on the phyllochron and the final leaf number. The (thermal) time interval separating the appearance of the flag leaf ligula and anthesis is less dependent on either genotype or environment than is the duration of the preceding stages (Amir and Sinclair, 1991). Final leaf number, the phyllochron, and the length of the interval between flag leaf ligula appearance and anthesis interval are considered as being the major determinants of development (Jamieson *et al.*, 1998). The duration of the various pre-anthesis stages can similarly be analysed in terms of final leaf number and the phyllochron, given the strong association between the length of time required to reach the terminal spikelet stage and the final leaf number (Jamieson *et al.*, 2007).

The current experiments were designed to characterize variability in the duration of the period between consecutive various pre-anthesis phases, the final leaf number, and the phyllochron in durum wheat (*Triticum turgidum* ssp. *durum*), with a focus both on identifying the nature of the genetic control over the duration of the various pre-anthesis phases, and on defining the environmental cues (day length and vernalization), if any, which underlie them. An additional focus was to determine whether the genotypic relationships between pre-flowering phases, final leaf number, phyllochron, and anthesis date are differentially affected by EPS, sensitivity to photoperiod, or sensitivity to vernalization.

## Materials and methods

### Plant materials and experimental design

The mapping population was a set of 100 recombinant inbred lines (RILs) bred from the cross cv. Ofanto (an early flowering, semi-dwarf cultivar released in 1990) × cv. Cappelli (a late flowering, tall, vernalization-requiring cultivar released in 1915). The experimental site was at Ottava, Sardinia (41° N 8° E; 225 metres above sea level). A set of similarly sized vernalized and nonvernalized seedlings was potted on 24 May (day length 14.8h), and a second sowing of vernalized seedlings on 23 December of the same year. Vernalization was achieved by imbibing the grain for 24h at room temperature, and then growing the seedlings in the dark at 4 °C for 40 days. Between 6 and 8 weeks below 5 °C is assumed to be sufficient for the full vernalization of most wheat cultivars (Davidson *et al.*, 1985; Griffiths *et al.*, 1985). Two pots (each containing three plants) were assigned to each RIL/treatment combination and were arranged in a completely randomized design. The May-sown plants (long days, vernalized plants: LDV, long days, nonvernalized: LD) were maintained outdoors and the December-sown ones (short days, vernalized) were kept in a greenhouse. The pots were watered and fertilized as required.

The LDV treatment was characterized by the least limiting conditions in both cold and day length, as confirmed by its mean transplanting–terminal spikelet period (TRA-TS) of 449 °Cd, close to the minimum of 400 °Cd proposed by Ritchie (1991) for fully vernalized wheat plants grown under long days. Differences in EPS among the RILs were therefore estimated from this treatment. The comparison between LDV and LD allowed for the separation of the effect of vernalization from that of photoperiod, while the comparison between LDV and SDV allowed for the separation of photoperiod from vernalization effects (Herndl *et al.*, 2008).

### Phenotyping

Although the timing of arrival at TS is most accurately assessed destructively, a simpler, nondestructive means of assay has been based on observing the timing of elongation of the first internode (Hay, 1978). The latter data were obtained by a twice-weekly measurement of the height above the ground of the ligule of the youngest fully emerged leaf on the main stem. The relationship between this height and the thermal time from TRA took the form of two linear segments of nonidentical slope: the shallower one reflected growth prior to stem elongation and the steeper one post stem elongation. The accumulated thermal time at this inflexion point was quantified using a ‘segmented regression’ approach, implemented in the split-line regression procedure within GENSTAT (2008). The plants were monitored on the same twice-weekly basis to allow the timing of the emergence of the penultimate leaf (PEN) and the flag leaf (FLA), booting (BOOT) and anthesis (ANT) to be recorded on the main stem. FLA, BOOT, and ANT correspond to stages 39, 45, and 65, respectively, of Zadoks *et al.* (1974). These timings defined the lengths of the TRA–TS, TS–ANT, FLA–ANT, PEN–ANT, and FLA–BOOT intervals, expressed in thermal time according to Weir *et al.* (1984), based on measured daily values of minimum and maximum temperature. The length of the photoperiod was based on the period between daybreak and when the sun had set 6 ° below the horizon (Weir *et al.*, 1984). Following Herndl *et al.* (2008) and White and Laing (1989), the relative response to vernalization (RRV) was computed from R (the inverse of the duration in °Cd of the various intervals) in the form 1 – (R_LD_/R_LDV_), and similarly RRP was given by 1 – R_SDV_/R_LDV_). These two indices enabled the quantification of photoperiod sensitivity and vernalization requirement in the form of deviations from the EPS response. The number and length of the leaves which had emerged on the main stem were recorded twice weekly until the flag leaf had become fully extended, following Haun (1973). A rate of leaf emergence was calculated for each plant from the slope of the regression between the Haun stage and the thermal time from TRA. Two separate regressions were performed for each plant: one included all the leaves and the other included only leaves 2–8, as recommended by Jamieson *et al.* (1995). For all plants, the linear regressions were both statistically significant, explaining >90% of the phenotypic variation. An average phyllochron (AvgPHY) was calculated as the reciprocal of the rate of leaf emergence obtained from the former regression, and a second phyllochron (PHY28) from the latter one. The total number of leaves borne by the main stem (LN_ANT_) and that of the leaves which had emerged by TS (LN_TS_) was recorded. The latter was calculated by substituting the thermal time elapsed from TRA to TS into the above regressions. The number of leaves which had emerged after TS (LN_afterTS_) was given by the difference LN_ANT_ – LN_TS_.

### Statistical treatment of phenotypic data

The magnitudes of the treatment, genotypic, and genotype × treatment interaction effects were obtained from a mixed-model analysis of variance (ANOVA) obtained by implementing the REML procedure within GENSTAT (2008). The same type of analysis was applied to each environment separately. The variance components and best linear unbiased predictors (BLUPs) related to each RIL and trait were calculated, and heritabilities were estimated from the resulting variance components on a line mean basis. The BLUPs were used to visualize frequency distributions across the set of RILs and to estimate genetic correlations, following Borràs-Gelonch *et al*. (2011).

### Quantitative trait locus mapping

Quantitative trait locus (QTL) analysis was performed using the software package MapQTL version 5.0 (van Ooijen and Voorips, 2004) and was based on the genotypic data and derived genetic map described by Marone *et al.* (2012) and Panio *et al.* (2013). Limit of detection (LOD) proﬁles obtained from simple interval mapping were used to identify the marker closest to each predicted QTL position, and this was then used as a cofactor to perform multiple QTL mapping analysis. LOD significance threshold levels were calculated via a 10 000 permutation test, provided within MapQTL. The length of the genetic interval between the flanking markers of each QTL was determined using the ΔLOD-1 support interval criterion. A number of QTL associated with a LOD scoring marginally below the significance threshold (LOD=3) were included only where they colocalized with one or more statistically significant QTL.

## Results

### Day length and temperature

**Fig. 1.**
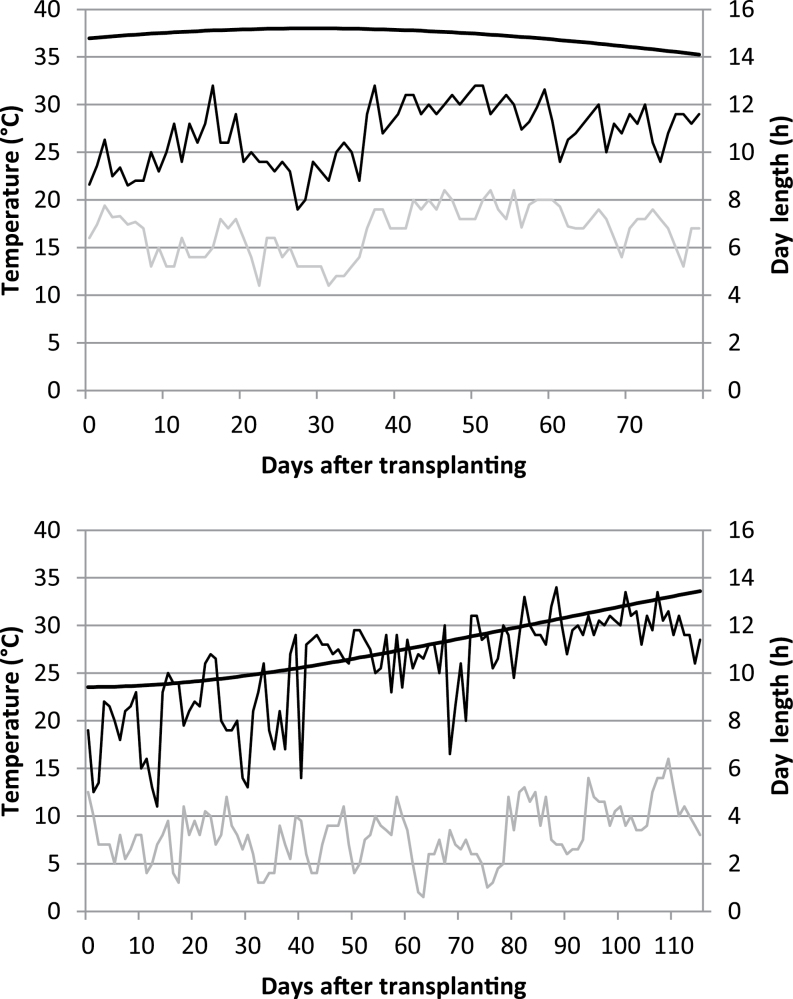
Maximum and minimum air temperature and the variation in day length between transplanting and anthesis in LDV- and LD-grown plants (top) and SDV-grown plants (bottom).

The photoperiod at the time of the May transplanting was 14.8h, while at the time of the December transplanting, it was 9.4h (Fig. 1). Following the May transplanting (LDV and LD), it peaked at 15.2h, around the mean of the time when the RILs had reached TS. At the mean anthesis date, the photoperiod was 15.1h for LDV-grown plants and 14.6h for the LD-grown ones. The SDV-grown plants experienced a lengthening photoperiod, reaching 10.3h around TS, 12.4h around FLA, and 13.1h around anthesis. The mean air temperature was about 20.3 °C over the TRA–TS interval for the LD- and LDV-grown plants, but only 13.9 °C for the SDV-grown ones. Over the period TS–ANT, the mean air temperature rose to 24.2 °C for the LDV-grown plants and to 18.1 °C for the SDV-grown plants.

### Trait analysis

The ANOVA derived from the full data set indicated the absence of a main genetic effect for most of the traits, but the presence of a major genotype × treatment interaction (Table 1). On the other hand, the genetic component was significant for most of the traits when each set of plants (LDV-, LD-, and SDV-grown) was analysed separately. The broad sense heritability was >90% with respect to the length of the TRA–ANT period for each set of plants, and less (but still high) for the duration of the other pre-anthesis stages. Comparable levels of heritability were observed for both LN and the phyllochron.

**Table 1.** ANOVA for and heritability of the various phenological traits expressed in the cv. Ofanto × cv. Cappelli RIL population

Trait	LDV			LD			SDV			Combined analysis			
	Genotype	Residual	h^2^	Genotype	Residual	h^2^	Genotype	Residual	h^2^	Genotype	GxE	Residual	h^2^
Anthesis (°Cd)	1138±179	438±32	92.6	22 951±3610	4118±439	94.2	3567±578	1490±117	91.4	147±579	8865±966	1639±78	4.4
TRA–TS (°Cd)	1472±243	1010±72	88.2	17 266±2593	1164±123	97.8	3270±639	4364±339	77.0	0±449	7522±848	2784±128	0.0
TS–ANT (°Cd)	268±65	824±61	61.1	3580±746	3583±386	74.0	8656±1411	3822±299	91.0	46±307	4070±518	3571±165	2.3
PEN–ANT (°Cd)	196±59	1004±72	49.4	1941±603	5715±582	49.5	290±79	1021±79	55.0	0±81	806±140	2054±96	0.0
FLA–BOOT (°Cd)	259±66	927±67	58.4	335±146	1723±177	36.5	355±84	874±68	64.3	150±45	165±45	1057±48	39.4
FLA–ANT (°Cd)	60±26	533±40	35.1	2363±577	3700±401	64.1	280±83	1190±93	51.1	0±71	680±125	2053±94	0.0
LN_ANT_ (no)	0.20±0.04	0.38±0.03	73.1	2.12±0.35	0.61±0.1	90.8	0.12±0.03	0.36±0.03	58.8	0.07±0.06	0.67±0.08	0.42±0.02	19.4
LN_TS_ (no)	0.12±0.02	0.16±0.01	78.6	0.89±0.15	0.34±0.0	87.6	0.11±0.02	0.22±0.02	67.7	0.00±0.03	0.36±0.04	0.22±0.01	0.0
LN_afterTS_ (no)	0.03±0.02	0.38±0.03	28.2	0.35±0.10	0.77±0.1	54.5	0.14±0.04	0.43±0.03	59.9	0.01±0.02	0.14±0.03	0.48±0.02	9.4
AvgPHY (°Cd)	5.74±2.80	66.1±4.6	30.4	21.42±5.47	41.2±4.2	61.1	53.9±8.89	26.5±2.1	89.9	2.07±2.32	21.8±3.57	47.3±2.2	12.0
PHY28 (°Cd)	5.39±3.33	87.1±6.0	24.7	16.79±7.52	95.8±9.4	34.9	15.1±4.29	60.4±4.6	52.8	4.4±2.1	7.01±2.77	79.5±3.6	22.2

Values are variance components±SE. Analysis was performed both within each treatment (LDV: long days, vernalized plants; LD: long days, nonvernalized plants; SDV: short days, vernalized plants) separately, and also on the combined data set. ANT: anthesis; AvgPHY: phyllochron relative to all the leaves BOOT: booting; FLA: flag leaf; LN: leaf number; PEN: penultimate leaf; PHY28: phyllochron relative to the leaves 2–8; TRA: transplanting; TS: terminal spikelet.

### Phenology

**Fig. 2.**
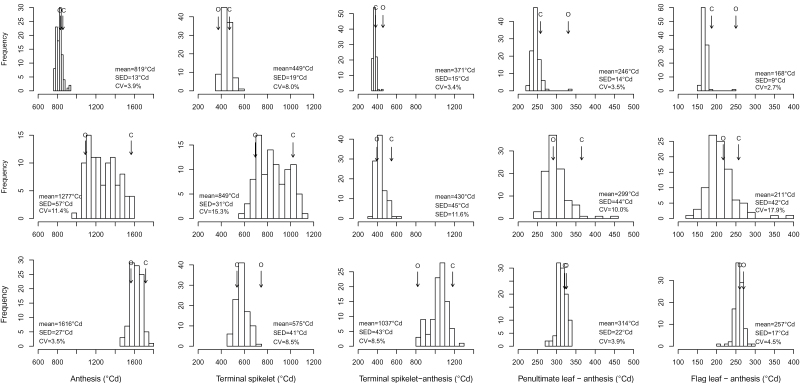
Distribution of RIL means (BLUPs, Best Linear Unbiased Predictors) for the lengths of the various pre-anthesis intervals. Top: long days, vernalized plants; middle: long days, nonvernalized plants; bottom: short days, vernalized plants. Arrows indicate the performance of the parents (O: cv. Ofanto; C: cv. Cappelli). CV: coefficient of variation; SED: standard error of the difference between BLUPs.

**Fig. 3.**
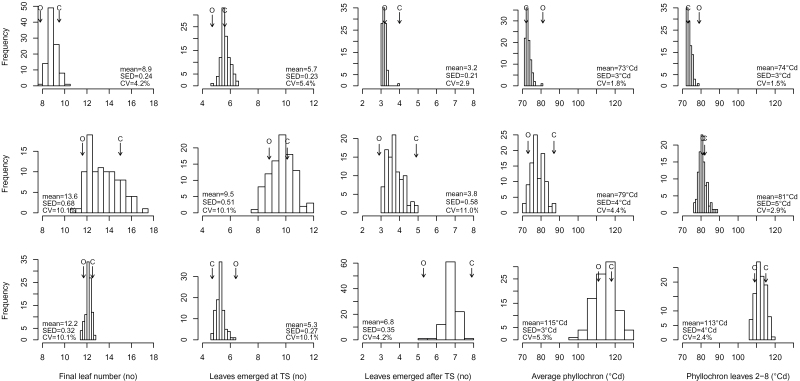
Distribution of RIL means (BLUPs, Best Linear Unbiased Predictors) for the number of leaves emerged prior to and after terminal spikelet and at anthesis, and for the two measured phyllochrons (relative to all the leaves and to the leaves from the second to the eighth). Top: long days, vernalized plants; middle: long days, nonvernalized plants; bottom: short days, vernalized plants. Arrows indicate the performance of the parents (O: cv. Ofanto; C: cv. Cappelli). CV: coefficient of variation; SED: standard error of the difference between best linear unbiased predictors.

The mean TRA–ANT period for the LDV-grown RILs was 42 days (equivalent to 819 °Cd), ranging from 40–48 days; Fig. 2). Across the set of RILs, the TRA–TS period was more variable than the TS–ANT one, and this stage represented about 50% of the whole pre-anthesis period. The mean LN_ANT_ was 8.9, with the lowest number (8) produced by cv. Ofanto (Fig. 3). More leaves were produced (mean 5.7) and the genetic variation was larger (5–6.5) before TS than after it. AvgPHY and PHY28 were very similar to one another, both with respect to their magnitude and their variation across the RIL population.

All LD-grown plants reached anthesis despite their lack of vernalization, but they flowered a mean of 22 days (457 °Cd) later than did the LDV-grown plants. The lack of vernalization had a large effect on the variation in the length of TRA–ANT interval among the RILs (29 days, 600 °Cd). The LD-grown plants needed almost twice the time to reach TS compared to the LDV-grown ones (43 vs. 22 days, 849 vs. 450 °Cd). The LD-grown material exhibited the most genetic variation with respect to the length of the TRA–TS interval (30–55 days, equivalent to 514 °Cd). The lengthened TRA–TS interval resulted in a reduction of the contribution of the TS–ANT interval to 34–36% of the whole pre-anthesis period. The PEN–ANT and FLA–ANT intervals were prolonged by, respectively, 50 and 40 °Cd, and genetic variation for these times became clear. The range in length of the PEN–ANT interval across the RILs was 252–365 °Cd (excluding two outliers), corresponding to 5 days, while that of the FLA–ANT interval varied from 132 to 395 °Cd (8–20 days). Nonvernalized plants developed many more leaves than did the vernalized ones; the range in LN_ANT_ across the LD-grown RILs was 11–17 (mean 14), while LN_TS_ ranged from 8 to 12 and LN_afterTS_ from 3 to 5. The mean values of AvgPHY and PHY28 were close to one another, and the difference between the LDV- and LD-grown RILs was greater for AvgPHY (ranging from about 70–90 °Cd) than for PHY28.

The SDV-grown plants took 796 °Cd longer than the LDV-grown ones to reach anthesis. The shorter photoperiod experienced by the SDV-grown plants strongly affected the length of the TS–ANT interval, inducing a mean increase of about 2.8-fold compared to the length of the same interval in the LDV-grown plants. The range in length of the TS–ANT interval among the RILs was 464 °Cd (30 days), and it comprised 45–64% of the whole pre-anthesis period. The FLA–ANT interval (258 °Cd) was longer than for either the LD- or LDV-grown plants. Only a narrow range in LN_ANT_ and LN_TS_ was recorded, and AvgPHY and PHY28 were each increased by about 30 °Cd compared to the plants grown under either LD or LDV. The clearest genetic variation for AvgPHY was displayed by the SDV-grown plants.

### Vernalization and photoperiod sensitivity

**Fig. 4.**
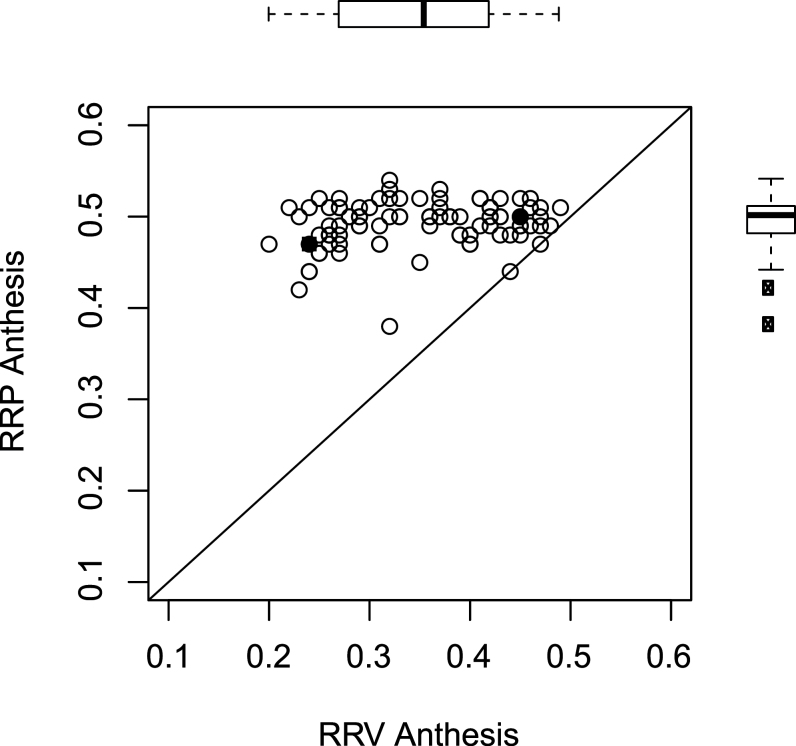
Sensitivity of RILs to photoperiod (RRP), estimated from anthesis date plotted against relative sensitivity to vernalization (RRV) and the corresponding boxplots. The RIL parents are indicated by either squares (cv. Ofanto) or circles (cv. Cappelli).

**Fig. 5.**
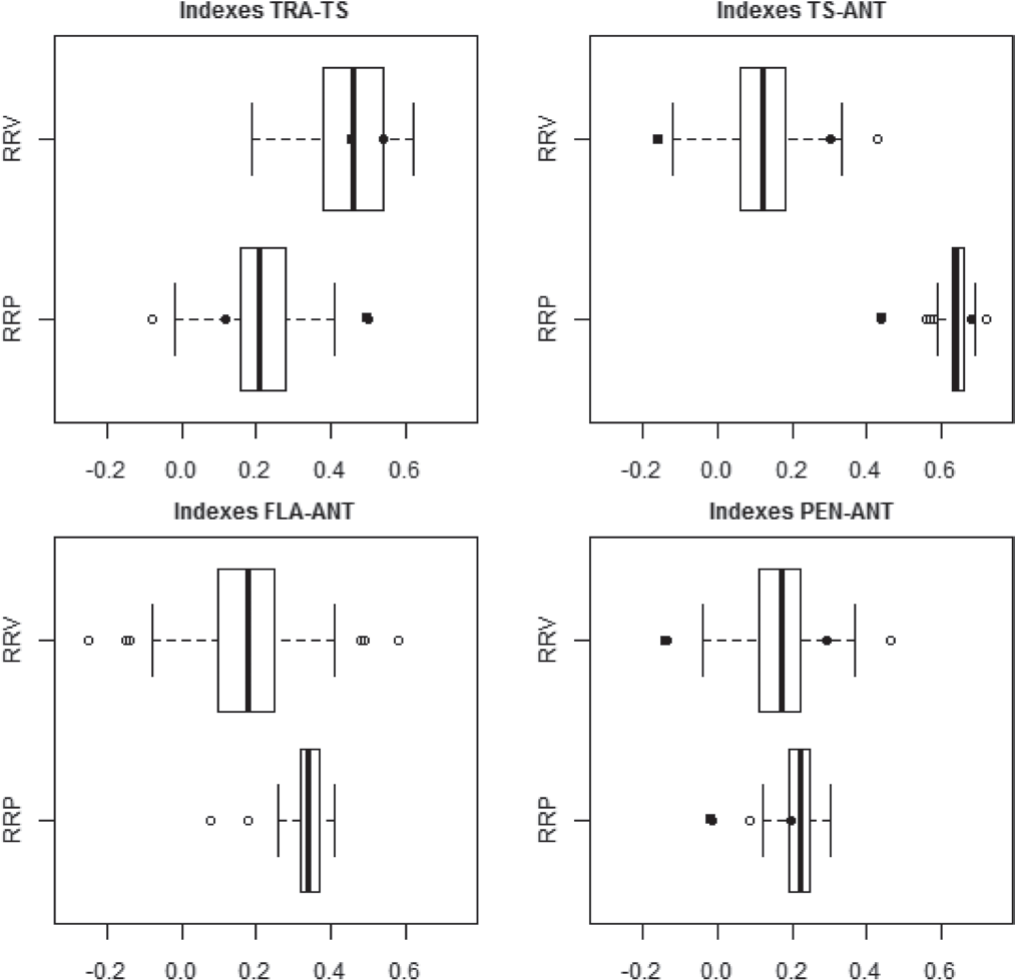
Boxplots illustrating the variation among the RILs with respect to relative photoperiod and vernalization sensitivity for the length of the various pre-anthesis intervals. The RIL parents are indicated by either squares (cv. Ofanto) or circles (cv. Cappelli).

The calculation of RRV and RRP allowed the effects of EPS on phenology to be recognized and thus made it possible to quantify the sensitivity of each RIL to vernalization and photoperiod. Across the whole population, the RRP for TRA–ANT was 0.49 (Fig. 4). In absolute terms this RRP value corresponded to a mean difference of 92 °Cd h^–1^ of day length between LDV (range 52–63 °Cd h^–1^) and SDV (range 133–160 °Cd h^–1^). RRV was lower but more variable than RRP, ranging from 0.20 to 0.49, as against 0.38–0.54. The two indices were correlated to one another, but only explained less than 6% of the overall variation (*R* ^2^=0.058, *P*<0.05). The triggering of most of the pre-anthesis stages was more sensitive to vernalization than to photoperiod (Fig. 5). The interval most strongly affected by vernalization, in terms of both mean effect and variability, was TRA–TS, but the FLA–ANT interval was also markedly affected. Photoperiod acted most strongly on the TS–ANT interval, although variability among the RILs was scarce. The TS–ANT interval was a mean of 70 °Cd h^–1^ of day length longer and more variable in the SDV plants (range 73–115 °Cd h^–1^) than in the LDV-grown ones (range 23–27 °Cd h^–1^).

### Genetic correlations

The observed genetic correlations between traits differed among the three treatments (Table 2). The traits most strongly correlated with the length of the TRA–ANT interval in the LDV-grown material were LN_TS_ (*R* ^2^=0.34, *P<*0.001), LN_ANT_ (*R* ^2^=0.36, *P<*0.001), and TS (*R* ^2^=0.61, *P<*0.001). The length of the TRA–TS interval was negatively correlated with that of TS–ANT, as was LN_TS_ with LN_afterTS_, and the phyllochron explained a lower (but nevertheless significant) proportion of the variation in time to reach anthesis, and was strongly correlated with the FLA–ANT and PEN–ANT phases. TS and LN_ANT_ were the main determinants of the length of the TRA–ANT interval for the LD-grown plants (*R* ^2^=0.79, *P<*0.001 and 0.66, *P<*0.001, respectively), while significant correlations also existed between the length of the TRA–ANT interval and all the traits except for PHY28. Among the LD-grown plants, there was no correlation between the length of the TRA–TS interval and any of the post-TS stages, while all three LN variables were positively correlated with AvgPHY (but not with PHY28). Finally, for the SDV-grown plants, both the length of the TS–ANT interval (*R* ^2^=0.46, *P<*0.001) and AvgPHY (*R* ^2^=0.34, *P<*0.001) were strongly associated with the length of the TRA-ANT interval. The length of the TRA–TS interval was negatively correlated that of the TS–ANT interval, as was LN_TS_ with LN_afterTS_.

**Table 2.** Genetic correlations among the measured phenological traits for LDV-, LD- and SDV- grown plants

	TRA–ANT	TRA–TS	TS–ANT	FLA–ANT	PEN–ANT	LN_ANT_	LN_TS_	LN_afterTS_	AvgPHY
Long days, vernalized plants									
TRA–TS	0.78***								
TS–ANT	0.05	–0.50***							
FLA–ANT	0.08	–0.28**	0.70***						
PEN–ANT	0.20*	–0.20*	0.65***	0.88***					
LN_ANT_	0.60***	0.69***	–0.26**	–0.33***	–0.36***				
LN_TS_	0.58***	0.77***	–0.45***	–0.38***	–0.30**	0.72***			
LN_afterTS_	0.09	–0.02	0.15	0.04	–0.12	0.45***	–0.17		
AvgPHY	0.24*	0.06	0.32**	0.44***	0.54***	–0.44***	–0.12	–0.47***	
PHY28	0.30**	0.22*	0.14	0.35***	0.44***	–0.32***	0.00	–0.47***	0.91***
Long days, nonvernalized plants									
TRA–TS	0.89***								
TS–ANT	0.53***	0.10							
FLA–ANT	0.42***	0.07	0.81***						
PEN–ANT	0.43***	0.11	0.77***	0.86***					
LN_ANT_	0.81***	0.91***	0.10	–0.01	0.02				
LN_TS_	0.67***	0.79***	–0.01	0.00	0.04	0.81***			
LN_afterTS_	0.63***	0.64***	0.21*	–0.02	–0.02	0.74***	0.26**		
AvgPHY	0.68***	0.63***	0.30**	0.17	0.23*	0.44***	0.32***	0.43***	
PHY28	0.17	0.11	0.16	0.21*	0.21*	–0.01	–0.03	0.04	0.47***
Short days, vernalized plants									
TRA–TS	–0.09								
TS–ANT	0.68***	–0.78***							
FLA–ANT	0.33**	–0.18	0.33***						
PEN–ANT	0.48***	–0.32***	0.54***	0.82***					
LN_ANT_	0.20	–0.07	0.19	–0.05	–0.08				
LN_TS_	–0.15	0.65***	–0.53***	–0.07	–0.13	0.16			
LN_afterTS_	0.27**	–0.55***	0.55***	0.02	0.03	0.66***	–0.63***		
AvgPHY	0.58***	–0.34***	0.62***	0.24*	0.50***	–0.40***	–0.23**	–0.14	
PHY28	0.31**	0.00	0.17	–0.04	0.12	–0.37***	–0.21*	–0.12	0.55***

ANT: anthesis; AvgPHY: phyllochron relative to all the leaves; BOOT: booting; FLA: flag leaf; LN: leaf number; PEN: penultimate leaf; PHY28: phyllochron relative to the leaves 2–8; TRA: transplanting; TS: terminal spikelet.

*P<0.05; **P<0.01; ***P<0.001.

### QTL analysis

**Fig. 6.**
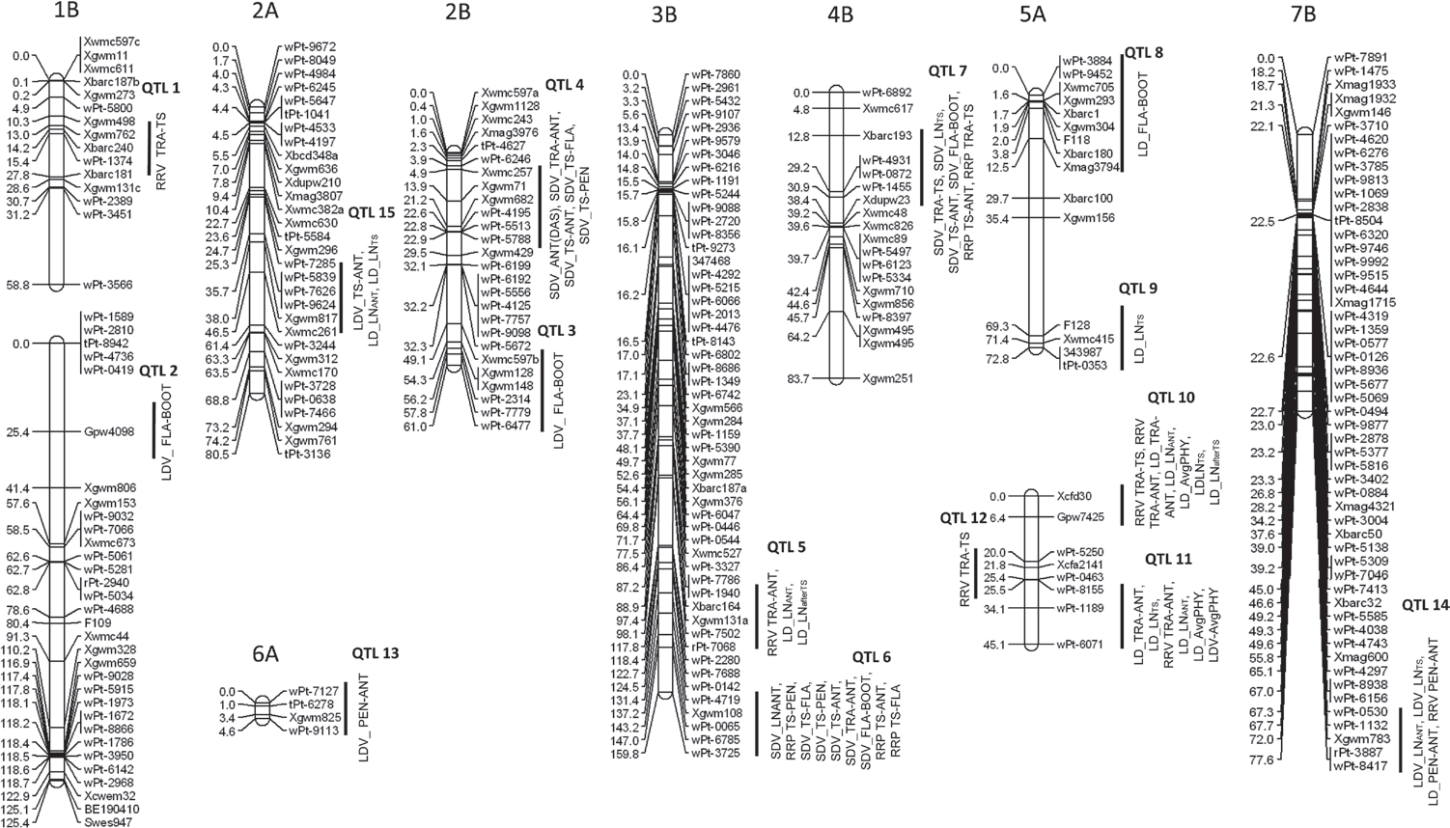
Chromosomal regions harbouring QTLs for phenological traits.

A total of 15 significant QTLs was identified, mapping to chromosomes 1B, 2A, 2B, 3B, 4B, 5A, 6A, and 7B (Table 3, Fig. 6). Variation for some of the traits was associated with a different spectrum of QTLs, depending on the treatment; for example, that for LN_ANT_ was determined by QTLs 5, 10, 11, and 15 in the LD-grown plants, by QTL 14 in LDV-grown ones, and by QTL 6 in the SDV-grown ones. Each locus (except for QTL 14) was specific to a treatment. Three loci underlay variation for the length of the TRA–ANT interval (QTLs 4, 5, 10, and 11); they were expressed by either the LD- or the SDV-grown plants, but not by the LDV-grown ones. The LOD values associated with these loci ranged from 4.5 to 12.3, and the proportion of the phenotypic variance explained (PVE) by each ranged from 17 to 46%. The two largest effect loci (QTLs 10 and 11) both mapped to chromosome arm 5AL and had a pleiotropic effect on some of the other traits. QTL 4 (mapping to chromosome arm 2BS) was the only locus for the TRA–ANT interval identified among the SDV-grown population, and QTL 5 (chromosome arm 3BL), identified in the LD-grown plants, the only one specific to the TRA–ANT interval. The latter locus was associated with a PVE of 36% and a LOD of 4.8.

**Table 3.** QTLs identified in each treatment

QTL	Chr.	Position (cM)	Peak marker	LDV	LD	SDV	LOD	*R* ^2^ (%)	Add. eff.
1	1BS	15.4	**wPt-1374**		**RRV (TRA–TS)**		3.00	9.6	–0.03
2	1BL	33.4	**Gpw4098**	**FLA–BOOT**			3.64	18.5	–6.05
	1BL	37.4	*Xgwm806*			*RRP (FLA–BOOT)*	2.16	11.1	0.06
3	2BS	54.3	**Xgwm128**	**FLA–BOOT**			3.16	10.8	–4.12
4	2BS	22.2	**Xgwm682**			**ANT (DAS)**	7.43	30.4	–1.25
	2BS	22.2	**Xgwm682**			**TRA–ANT**	4.59	17.5	–25.03
	2BS	21.2	**Xgwm682**			**TS–ANT**	3.34	11.8	–30.00
	2BS	21.2	**Xgwm682**			**TS–FLA**	3.00	9.6	–24.70
	2BS	21.2	*Xgwm682*			*TS–PEN*	2.85	9.2	–22.72
	2BS	22.0	*Xgwm682*	*LN*_*TS*_			2.62	10.1	–0.10
	2BS	37.3	*wPt-5672*			*TRA–ANT*	2.54	12.1	–22.54
5	3BL	107.1	**rPt-7068**		**RRV (TRA–ANT)**		4.79	36.3	–0.05
	3BL	97.0	**Xgwm131a**		**LN**_**ANT**_		3.28	7.2	–0.42
	3BL	106.1	*wPt-7502*		*LN*_*afterTS*_		2.71	11.8	–0.16
6	3BL	147.0	**wPt-6785**			**LN**_**ANT**_	3.29	14.6	0.10
	3BL	142.2	**wPt-0065**			**RRP (TS–PEN)**	3.18	13.2	0.01
	3BL	143.2	**wPt-0065**			**TS–FLA**	3.14	10.4	25.75
	3BL	143.2	**wPt-0065**			**TS–PEN**	3.08	10.1	23.80
	3BL	143.2	*wPt-0065*			*TS–ANT*	2.64	9.2	26.52
	3BL	140.2	*wPt-0065*			*TRA–ANT*	2.53	7.7	16.96
	3BL	128.5	*wPt-0142*			*FLA–BOOT*	2.39	10.6	5.06
	3BL	142.2	*wPt-0065*			*RRP (TS–ANT)*	2.37	9.9	0.01
	3BL	140.2	*wPt-0065*			*RRP (TS–FLA)*	2.33	10.7	0.01
7	4BS	29.2	**wPt-4931**			**TRA–TS**	4.22	18.3	24.89
	4BS	22.8	*wPt-4931*			*LN*_*TS*_	2.85	20.1	0.17
	4BS	29.2	*wPt-4931*			*TS–ANT*	2.81	9.8	–32.51
	4BS	27.8	*wPt-4931*			*RRP (TS–ANT)*	2.28	10.3	–0.01
	4BS	37.9	*Xdupw23*			*FLA–BOOT*	2.25	8.8	–5.34
	4BS	26.8	*wPt-4931*			*RRP (TRA–TS)*	2.16	11.2	0.04
8	5AS	2.0	**F118**		**FLA–BOOT**		3.00	13.6	–4.32
9	5AL	72.8	**343987**		**LN**_**TS**_		3.00	7.4	0.26
10	5AL	0.0	**Xcfd30a**		**RRV (TRA–TS)**		13.73	58.0	–0.08
	5AL	0.0	**Xcfd30a**		**RRV (TRA–ANT)**		12.31	45.9	–0.06
	5AL	0.0	**Xcfd30a**		**TRA–ANT**		10.45	37.5	–105.64
	5AL	0.0	**Xcfd30a**		**LN**_**ANT**_		8.59	28.0	–0.88
	5AL	0.0	**Xcfd30a**		**AvgPHY**		6.78	27.9	–1.99
	5AL	0.0	**Xcfd30a**		**LN**_**TS**_		6.67	25.9	–0.49
	5AL	0.0	**Xcfd30a**		**LN**_**afterTS**_		3.07	13.2	–0.18
11	5AL	45	**wPt-6071**		**TRA–ANT**		5.03	18.6	–72.35
	5AL	45.1	**wPt-6071**		**LN**_**TS**_		4.56	18.7	–0.42
	5AL	39.1	**wPt-1189**		**RRV (ANT)**		4.45	17.5	–0.04
	5AL	45.0	**wPt-6071**		**LN**_**ANT**_		3.48	13.0	–0.59
	5AL	34.0	*wPt-1189*	*AvgPHY*			2.90	14.2	0.53
	5AL	39.1	*wPt-1189*		*AvgPHY*		2.49	11.2	–1.25
12	5AL	21.8	**Xcfa2141**		**RRV (TRA–TS)**		**3.45**	13.6	–0.04
13	6AS	1.0	**tPt-6278**	**PEN–ANT**			**3.3**	14.5	–0.93
14	7BL	70.0	**Xgwm783**	**LN**_**ANT**_			**4.11**	19.0	–0.18
	7BL	69.7	**Xgwm783**		**RRV (PEN–ANT)**		**3.18**	15.3	–0.03
	7BL	77.0	**rPt-3887**	**LN**_**TS**_			**3.10**	13.7	–0.13
	7BL	69.7	*Xgwm783*		PEN–ANT		*2.39*	10.0	–9.99
15	2AS	35.0	**wPt-9624**		**LN**_**ANT**_		**3.00**	10.0	0.51
	2AS	38.0	*Xgwm817*	*TS–ANT*			2.07	8.9	–3.97
	2AS	46.5	Xwmc261c		LN_TS_		2.32	7.9	0.26

Bold indicates those associated with LOD ≥3; italic indicates those associated with LOD <3. Add. eff.: Additive effect of the Ofanto allele; ANT: anthesis; AvgPHY: phyllochron relative to all the leaves; LDV: long days, vernalized plants; LD: long days, nonvernalized plants; SDV: short days, vernalized plants; LOD: limit of detection; BOOT: booting; FLA: flag leaf; LN: leaf number; PEN: penultimate leaf; RRP: response to photoperiod; RRV: response to vernalization; TRA: transplanting; TS: terminal spikelet.

The duration of the TRA–TS interval was under the genetic control of four QTLs, of which three (QTL 1 on chromosome arm 4BS, QTL 7 on 1BS, and QTL 12 on 5AL) acted specifically on the length of this interval. The strongest effect was exerted by QTL 7 (PVE of nearly 20% for the SDV-grown plants), compared with QTL 1 (PVE of 10%) and QTL 12 (14%). QTL 10 also acted strongly on the length of this interval (PVE of 58%). Other significant QTLs were mapped to chromosome arms 2BS (QTL 4) and 3BL (QTL 6) for the length of the TS–ANT, TS–FLA, and TS–PEN intervals, each associated with a PVE of around 10%. The loci underlying the length of the FLA–BOOT interval were QTL 2 on chromosome arm 1BL, QTL 3 on chromosome arm 2BS, and QTL 8 on chromosome arm 5AS; while those for the length of the PEN–ANT stage were QTL 13 on chromosome arm 6AS and QTL 14 on chromosome arm 7BL. These loci were detected among either the LDV- or LD-grown plants and were associated with a PVE of 14–15%.

### QTLs controlling leaf number and the phyllochron

The largest effect locus affecting LN throughout the life cycle was QTL 10 (chromosome arm 5AL). This locus was also the only one which influenced both the phyllochron and LN_afterTS_. Variation in LN_TS_ was contributed by QTL 9 (chromosome arm 5AL), while LN_ANT_ was under the control of five loci (QTLs 5, 6, 11, 14, and 15) mapping to, respectively, chromosome arms 3BL, 3BL, 5AL, 7BL, and 2AS. The only one of these loci which was specific for just LN_ANT_ was QTL 15. A minor effect on AvgPHY, detected both among the LDV- and the LD-grown plants, was exerted by QTL 11; in both cases the LOD fell below the threshold of 3 (2.9 and 2.5) and PVEs were 14 and 11%, respectively.

### Expression of most QTLs was treatment specific

Five of the 15 QTLs were detectable among the LDV-grown plants, of which three (QTLs 2, 3, and 13) specified variation in the length of either the FLA–BOOT or PEN–ANT intervals and were detected in neither the LD- nor the SDV-grown materials.

The highest number of QTLs was identified among the LD-grown plants. Seven of these nine QTLs were not detected in either of the other two sets of material, and two (QTLs 5 and 10) were associated with large PVEs for the length of the TRA–ANT interval, LN_ANT_, and LN_TS_. The SDV-grown plants produced the fewest QTLs (three in total: QTLs 4, 6, and 7), none of which were detectable in either the LD- or the LDV-grown materials. The QTLs involved were mainly associated with the control of the lengths of the TRA–ANT interval and the post-TS stages.

## Discussion

The RIL population displayed variation for the length of each of the individual pre-anthesis intervals, reflecting the divergent phenology of the parental cultivars: cv. Cappelli is a late-flowering, highly photoperiod sensitive type with a substantial vernalization requirement (Motzo and Giunta, 2007), while cv. Ofanto is relatively recent constitution, earlier than Cappelli when sown in autumn (Panio *et al.*, 2013). The population was also expected to segregate with respect to EPS, since modern Italian cultivars such as cv. Ofanto harbour much less effective alleles for this trait than do the older traditional types such as cv. Cappelli (Motzo and Giunta, 2007). Nevertheless, the indication was that the contribution of genotype to the global variance in phenotype was low for most of the traits. This apparent anomaly was, however, balanced by the large contribution made by the genotype × treatment interaction. The interpretation is that, although the same lines were analysed in each of the three treatments, phenology was being determined by a different set of genes in each case: in the LDV-grown plants only genes controlling EPS were active, in the LD-grown ones both EPS and vernalization requirement ones were involved, while in the SDV experiment the relevant genes were those responsible for EPS and for photoperiod sensitivity. This assumption is consistent with the outcome of the QTL analysis, since a different spectrum of loci emerged from each experiment.

Some of the QTLs identified here map to genomic regions known to harbour loci affecting heading date in bread wheat. For example, the chromosome 1B region, in which QTL 1 was mapped, is similar to the one on harbouring loci specifying heading date in bread wheat (Griffiths *et al.*, 2009; Reif *et al.*, 2011). The site of QTL 2 probably overlaps the stem elongation QTL identified by Borràs-Gelonch *et al*. (2011), as does that of QTL 14 with an ear emergence QTL described by Griffiths *et al.*, (2009). QTLs 3, 5, 9, and 13 similarly match the map position of loci specifying ear emergence (Griffiths *et al.*, 2009), while QTLs 3, 4, 8, and 10 map to matching regions identified by Hanocq *et al.* (2007) as sites of heading date QTLs.

### EPS QTLs

Rather few loci affecting EPS were identified, and the size of the effect of those which were identified was smaller than those associated with sensitivity to either photoperiod or vernalization. Major genes controlling EPS have only rarely been reported (Snape *et al.*, 2001), presumably because of the difficulty of designing experiments in which their action is not confounded by other classes of gene (Laurie, 1997; Griffiths *et al.*, 2009). Based on the length of the TRA–ANT interval, no EPS QTLs were apparent; instead, they were only detected by dividing the pre-anthesis development of the plants into discrete phases and by considering LN and the phyllochron. The LDV-grown plants were designed to identify EPS genes, since they avoided exposure to either low temperatures or short day lengths. The extent of the genetic variation caused by EPS genes with respect to the length of the TRA–ANT interval was similar to that noted by van Beem *et al.* (2005) in their comparison of 51 cultivars. Nevertheless, it was clear that these genes affected both LN_ANT_ and LN_TS_ and, to a lesser extent, the phyllochron. EPS genes have been proposed to control how many leaf or spikelet primordia are initiated (Gotoh, 1977; Hoogendoorn, 1985). QTL 14 was a determinant of both LN_ANT_ and LN_TS_, and its location on chromosome arm 7BL fits the conclusions drawn by Flood and Halloran (1983) and Hoogendoorn (1985) regarding the site of EPS genes in bread wheat. Similarly, the location of QTL 11 may match that of EPS genes described by Kato *et al.* (2003), since its PVE was 14%. Its strong effect confirms that the action of EPS genes is also exerted via the control of leaf appearance rate. A role of the phyllochron in the expression of EPS has also recently been suggested (He *et al.*, 2012). The two other EPS-specific QTLs identified mapped to chromosome arms 1BL and 2BS. EPS genes are known to reside on chromosome 2B (Scarth and Law, 1983; Shindo *et al.*, 2003). The latter authors have suggested that a EPS QTL lying close to the major *Ppd-B1* (photoperiod sensitivity) gene may represent the orthologue of the barley gene *eps2S* (Laurie *et al.*, 1995).

### QTLs associated with vernalization requirement

The RRV associated with the length of the TRA–ANT interval was very consistent with that reported by Herndl *et al.* (2008) in a study of 26 European bread wheat cultivars. The RIL parent cv. Cappelli has a high vernalization requirement, while even current Italian spring type cultivars such as cv. Ofanto have a residual requirement (Motzo and Giunta, 2007). Winter cultivars carry one or more of the major *Vrn* (vernalization requirement) genes, which have a large effect on the length of the sowing to TS interval (Griffiths *et al.*, 1985; Robertson *et al.*, 1996; Motzo and Giunta, 2007). The behaviour of the RILs suggested that much of the variation in the length of the TRA–TS interval, of the TS RRV, and in LN_ANT_ was the principal driver of variation in the length of the TRA–ANT interval and that this variation reflected genetic differences amongst the RILs with respect to their individual vernalization requirement. The time required to reach TS was the trait most often associated with the QTLs detected in the LD-grown plants. The colocation of QTLs controlling the length of the TRA–TS interval and LN_TS_ (QTLs 10 and 11) should be viewed in the light of the much discussed physiological association between the length of the period during which primordia are produced and leaf number (Kirby, 1990; Brooking and Jamieson, 2002; Jamieson *et al.*, 2007). The degree of variability in LN_ANT_ which was induced by the lengthening of the TRA–TS interval was mirrored in the LD-grown plants by a positive association between LN_ANT_ and AvgPHY, likely reflecting the deceleration in the rate of appearance of the leaves later than the eighth (Miglietta, 1991). As a consequence, AvgPHY (but not PHY28) was associated with many of the phenological events occurring both before and after TS. The implication is that vernalization requirement genes extend their effect beyond TS. The association between LN_ANT_ and AvgPHY resulted in the pleiotropic action of QTL 10 on AvgPHY, establishing a genetic basis for phyllochron variation when the leaf number exceeds eight. The *Vrn-A1* locus is located on chromosome arm 5AL (Galiba *et al.* 1995), where four of the QTLs detected only in the LD-grown material were located. The ability of every RIL to flower, despite the absence of any vernalization treatment and the imposition of a long-day photoperiod, implied that none of the RILs carried the winter type (recessive) allele at either *Vrn-A1* or *-B1.* Based on the allele present at the microsatellite locus *Xcfa2141* linked to QTL 12, the indication is that QTL 12 is identical to *Vrn-A1*. Vernalization-responsive QTLs were also located on chromosome arms 1BS, 3BL, and 7BL, sites which are consistent with the map locations of *Vrn-2* and *Vrn-B3* (Goncharov, 2003; Kato *et al.*, 2003; Yan *et al.*, 2006).

### QTLs associated with photoperiod sensitivity

The RILs displayed only a limited extent of variation for the length of the TRA–ANT interval when grown under short-day conditions. Most the variation present was associated with AvgPHY and the length of the TS–ANT interval, the variation in length of which was partly explicable by variation in the phyllochron. The notion that the prolonged sensitivity of wheat plants to photoperiod after TS can affect the phyllochron is in line with conclusions drawn by both Gonzáles *et al*. (2005b) and Miralles and Richards (2000). Two of the three QTLs identified (QTLs 4 and 6) controlled several substages within the TS–ANT interval, underlining the importance of the length of the TS–ANT interval for the expression of photoperiod sensitivity (Fig. 2). QTL 4 could correspond to some *Ppd-B* genes that have already been mapped on chromosome 2B (Hanocq *et al.* 2007). In particular, the presence of a common DArT marker (*wPt5672*) reported by Crossa *et al.* (2007) and Le Gouis *et al.* (2012) on chromosome 2B confirmed the proximity to the *Ppd-B1* gene. The low variability of LN_ANT_ could be attributed to a low level of genetic variation in the timing of the response to day length in the RIL population (i.e. in the time of the final commitment of the flag leaf primordium). The initiation of the last leaf primordium can occur at any point up to and beyond TS, which provides one of the ways in which genetic polymorphisms in photoperiod sensitivity can be translated into variation in LN and hence in anthesis date (Brooking *et al.*, 1995). On the other hand, the RIL population diverged markedly with respect to LN_afterTS_, resulting in a negative correlation between the number of leaves which emerged before and after TS, and this was in turn responsible for the strong negative correlation between the lengths of the two consecutive intervals TRA–TS and TS–ANT. The cv. Ofanto allele at QTL 7 delayed TS while simultaneously reducing the length of the TS–ANT interval. In contrast, Borràs-Gelonch *et al*. (2011) have shown that in bread wheat, there was a positive genetic correlation between the time taken to reach TS and the length of the TS–ANT interval; the underlying cause of this difference in behaviour is hard to discern, as LN_ANT_ was not monitored in the current experiment. The only way in which an almost constant LN_ANT_ can result in variation in LN before and after TS is where there is variation in the time elapsed between the commitment to the formation of the flag leaf primordium and the initiation of the TS primordium at the meristematic apex, reflecting genetic variation with respect to photoperiod sensitivity during this period (Rawson, 1970). The remarkable impact of altering the length of the TS–ANT interval must therefore represent an outcome of a magnifying effect of the high phyllochron on the number of leaves which emerge after TS. The lengthening of the TS–ANT interval induced by short-day conditions was accompanied by a lengthening of the PEN–ANT interval, implying that the period of maximum spike growth can be positively affected by the expression of genes controlling photoperiod sensitivity. In the present case, however, the extent of the variation was insufficient to enable the detection of QTLs specifically controlling the length of this stage under short-day conditions.

### Conclusions

The splitting of the period between planting and anthesis into a series of physiologically based components has allowed the detection of many more genetic factors responsible for anthesis date than would have been achieved by simply searching for QTLs associated solely with anthesis date. At the same time, manipulating the environment in which the plants were grown to isolate known flowering cues has given the opportunity to define how each of the resulting QTLs identified interact with the growing environment. As well as the well-recognized impact of vernalization on plant developmental prior to TS, it was also possible to show that the vernalization effect can extend well beyond TS, due to its impact on LN and the phyllochron. EPS genes appear to affect the lengths of the pre-anthesis intervals via their independent effect on both LN and the phyllochron. For the particular RIL population in question, the length of the TRA–TS interval was correlated with that of the TRA–ANT interval only where the anthesis date was dependent on the final LN. Otherwise, it was the length of the TS–ANT interval which was the most strongly correlated with the timing of anthesis. Under an inductive photoperiod regime, the extent of genetic variation for the length of the pre-anthesis intervals was more marked than for anthesis date itself, due to the photoperiod sensitivity of the TRA–TS interval, coupled with a limited effect on total LN. The implication is that similar anthesis dates could potentially be arrived at even though the lengths of the various pre-anthesis intervals varied.

The overall result of the experiments supports the idea that, although intensive breeding and selection over the past century has succeeded in reducing the length of time between sowing and flowering of the durum wheat, there remains potential to manipulate the duration of the various pre-anthesis stages; the recognition of the genetic basis of these durations via the identification of relevant QTLs could lead to a marker-based strategy for fine-tuning the crop to its growing environment.
